# Mechanical and structural characterisation of the dural venous sinuses

**DOI:** 10.1038/s41598-020-78694-4

**Published:** 2020-12-10

**Authors:** Darragh R. Walsh, James J. Lynch, David T. O’ Connor, David T. Newport, John J. E. Mulvihill

**Affiliations:** 1 Bernal Institute, University of Limerick, Limerick, Ireland; 2 School of Engineering, University of Limerick, Limerick, Ireland; 3 Health Research Institute, University of Limerick, Limerick, Ireland

**Keywords:** Nervous system, Materials science, Biomedical engineering, Electron microscopy, Histology

## Abstract

The dural venous sinuses play an integral role in draining venous blood from the cranial cavity. As a result of the sinuses anatomical location, they are of significant importance when evaluating the mechanopathology of traumatic brain injury (TBI). Despite the importance of the dural venous sinuses in normal neurophysiology, no mechanical analyses have been conducted on the tissues. In this study, we conduct mechanical and structural analysis on porcine dural venous sinus tissue to help elucidate the tissues’ function in healthy and diseased conditions. With longitudinal elastic moduli values ranging from 33 to 58 MPa, we demonstrate that the sinuses exhibit higher mechanical stiffness than that of native dural tissue, which may be of interest to the field of TBI modelling. Furthermore, by employing histological staining and a colour deconvolution protocol, we show that the sinuses have a collagen-dominant extracellular matrix, with collagen area fractions ranging from 84 to 94%, which likely explains the tissue’s large mechanical stiffness. In summary, we provide the first investigation of the dural venous sinus mechanical behaviour with accompanying structural analysis, which may aid in understanding TBI mechanopathology.

## Introduction

The dural venous sinuses are large venous conduits within the dura mater layer of the meninges that are responsible for draining virtually all of the venous blood from the cerebral hemispheres^1^. Thus, their injury or acute occlusion can lead to significant clinical complications^2^ and is considered a life-threatening injury^3^. The sinuses are implicated in a myriad of diseases^4^ and their laceration in severe head injuries is a significant clinical concern^5^. Traumatic brain injury (TBI) represents an ever increasing public health and societal burden^6^. According to estimates by the Centers for Disease Control and Prevention, up to 1.7 million TBIs occur annually in the United States and 30.5% of all injury-related deaths are associated with TBI^7^. Efforts to reduce the incidence and societal burden of TBI rely on improving our understanding of the injury’s pathological cascade^8,9^. However, there is still much debate about the mechanistic and structural role of many intracranial tissues during TBI including the pia-arachnoid complex^10^, the dura mater^11^, the falx cerebri and tentorium cerebelli^12^, and indeed the dural venous sinuses.

**Figure 1:**
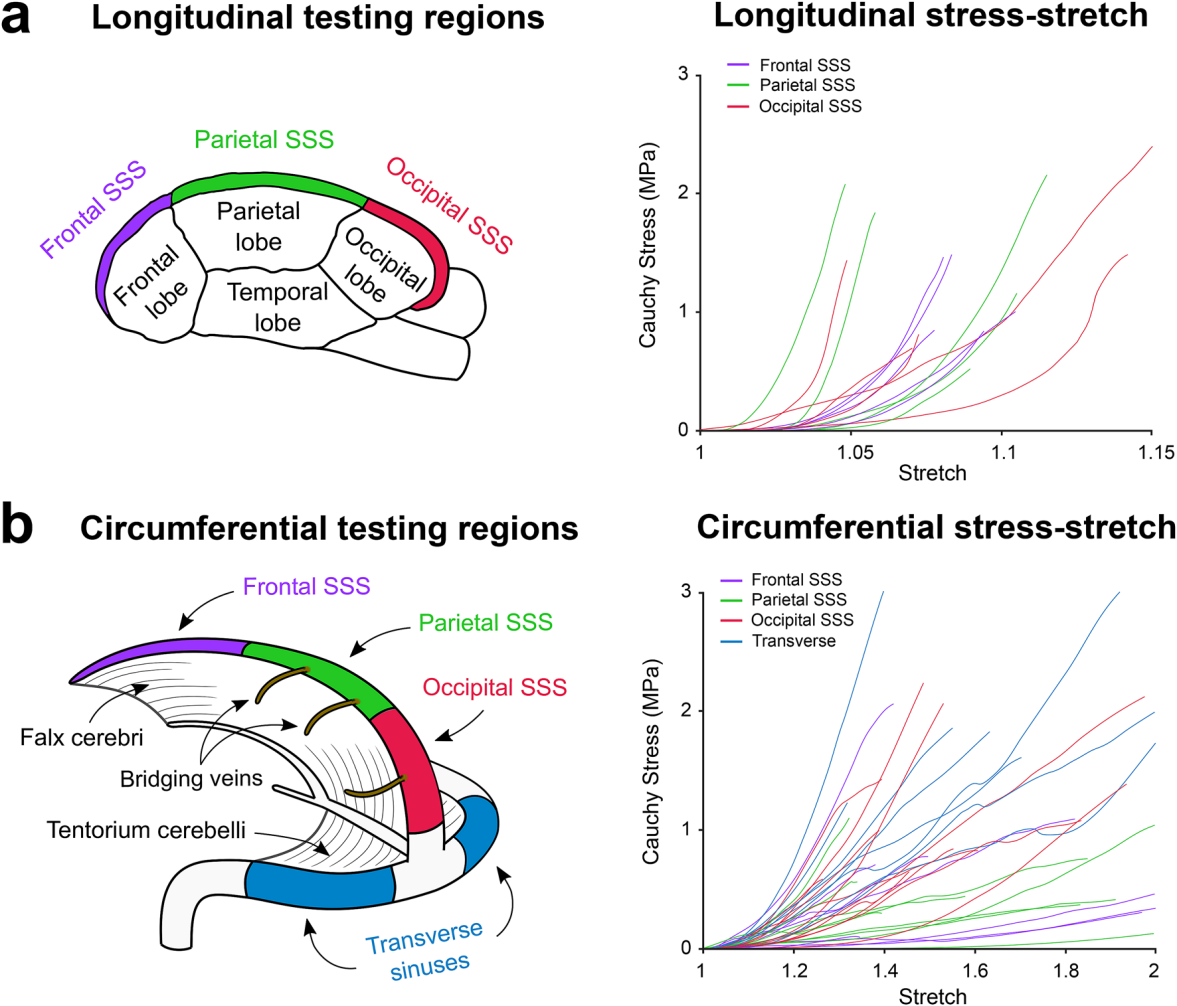
Illustration of the regions tested with the accompanying plots of the Cauchy stress-stretch curves of the dural venous sinus samples tested in (**a**) the vessel’s longitudinal test direction and (**b**) the vessel’s circumferential test direction. Note the different scales on the x-axes of the stress-stretch graphs.

There are numerous dural venous sinuses present throughout the cranial cavity including the superior sagittal sinus (SSS), the transverse sinus, the inferior sagittal sinus and the straight sinus (see Fig. 1b). The dural sinuses occupy a significant volume within the cranial cavity. In humans, the largest of the sinuses, the SSS, has a length of $\approx$ 25 cm^13^ and a lumen diameter ranging from 4 to 10 mm^14^. The SSS is connected to the frontal and parietal bones superiorly which leads to the development of an impression on the cranial bones^15^. At its inferior edge, the SSS is connected to the falx cerebri. The falx is an extension of the dura mater which runs within the longitudinal fissure of the brain and partitions the cerebral hemispheres. Similarly, another extension of the dura mater, the tentorium cerebelli runs in the lateral fissure of the brain and is posteriorly connected to the transverse sinuses (see Fig. 1b). Computational analyses exploring the role of the falx and tentorium in influencing brain strains during TBI have identified that these stiff, fibrous tissues have a significant influence on the stress-strain response of the brain during dynamic impacts^12,16–18^. As the falx and tentorium are connected to the SSS and transverse sinuses, the biomechanical response of the sinuses likely influences the in vivo biomechanical response of the falx and tentorium. Thus, quantifying the currently unknown mechanics of the dural sinuses is desirable for advancing the brain strain prediction accuracy of current FE head models^9^.

The rapid acceleration of the head associated with TBI results in the propagation of deleterious stresses and strains to intracranial tissues^19,20^. This results in a host of secondary injuries such as localised bruising and haemorrhaging of tissues. Acute subdural haematoma (ASDH) is a frequent co-morbidity of severe TBI characterised by a haemorrhage between the dura and arachnoid mater membranes^21^. ASDH is associated with extremely low functional recovery rates of approximately 19%^22^. Due to their anatomical location, the dural venous sinuses are also implicated in the mechanopathology of TBI-mediated ASDH. A network of bridging veins transport deoxygenated blood from the brain, across the subdural space, to the SSS. Rupture of these bridging veins accounts for approximately one third of all cases of ASDH^23^. Efforts to identify the threshold for TBI-mediated ASDH have, in part, relied on FE models with biofidelic representations of the bridging vein—SSS complex^24^. Studies suggest that understanding the anatomical connection between the bridging veins and the SSS, and indeed the structural architecture of both tissues, is of importance in elucidating the etiopathology of ASDH^9,25,26^. However, despite the complex anatomical connection between the SSS and the bridging veins^27^ and accordingly, the propensity for the SSS to influence the stress-strain distribution within bridging veins during TBI events^25^, no in-depth mechanical and structural evaluations of the SSS have been published in the literature.

From a clinical perspective, the sinuses are susceptible to pathological luminal narrowing as a result of cerebral venous sinus thrombosis (CVST)^28^. CVST is a rare but potentially life-threatening condition, with a mortality rate of roughly 10% in those affected^29^. In 70% of cases, the thrombosis occurs in either the SSS or transverse sinus^30^. With one in three patients with CVST suffering from intracerebral haemorrhage^31^, quickly regaining sinus patency is a clinical priority. Microcatheter balloons designed for coronary angioplasty procedures have been utilised to treat sinus pathologies^32^. The potentially significant mechanical mismatch between coronary vessels and the dural venous sinuses could result in clinicians inadvertently causing permanent damage to the sinuses^33^. Thus, a comparison between the sinus tissue’s circumferential mechanical response, a proxy to the vessel’s inflation behaviour, with the tissues these angioplasty devices are typically deployed in is warranted.

In this study, we present the first mechanical evaluation of the dural venous sinuses with accompanying structural and geometrical analysis to improve understanding on the role of these tissues in the etiology of TBI and TBI-mediated ASDH. Further, we provide a comparison to coronary vessels toward aiding the design of prospective medical devices for treating CVST.

## Materials and methods

### Tissue acquisition and preparation

Heads from 12 pigs (7 months old, gender unknown) were taken from a local abattoir following CO$_2$ euthanasia on site (Rosderra Meats, Nenagh, Ireland). Animal sacrifice was conducted in line with normal abbatoir guidelines and was not altered for this study. Heads were dissected within 4 h of animal euthanasia. Dura mater tissue containing the dural venous sinuses was dissected from the heads as described previously^34^. The sinuses were then isolated from the dura mater tissue. Sinus segments were frozen to $-20 \,^{\circ }$C in cryoprotectant media to prevent mechanical and structural degradation of the tissue^35^. The utilised cryoprotectant media contained Dulbecco’s modified Eagle’s medium (DMEM) as a vehicle solution to which the cryoprotectant agents dimethyl sulfoxide (DMSO) (at 1.8 M concentration) and sucrose (at 0.1 M concentration) were added^35^. Research on both skin^36^ and arterial^37^ tissues has demonstrated that cryopreservation with cryoprotectant media has a negligible effect on the mechanical response of both tissues. Samples were frozen for no more than 4 weeks prior to thawing in a water bath at $37\,^{\circ }$C^35^. Frozen tissue sections took, on average, approximately 30 minutes to fully thaw.

Thawed samples were then dissected utilising a scalpel for mechanical and structural analysis. The sinuses were sectioned into 4 regions of interest (see Fig. 1b for illustration). The first 3 regions of interest were the segments of the SSS. The SSS was sectioned into 3 segments; the frontal, parietal and occipital regions. These regions were defined in relation to the subjacent lobes of the cerebrum. The division between the frontal and parietal lobes for each SSS was identified by the position of the cruciate sulcus on the porcine brain, which has been homologised to the central sulcus in humans^38^. This point was marked on the SSS using specialised biological tissue dye (CDI tissue staining dye) prior to complete excision of the SSS from the cranial cavity to allow for identification of the frontal and parietal SSS regions. The occipital specimens were acquired from the posterior portion of the SSS adjacent to the confluence of the sinuses. The fourth region of interest was the tissue associated with the transverse sinuses (which is located adjacent to the lateral edges of the confluence of the sinuses). Samples from the SSS were prepared for either testing in the circumferential direction utilising ring testing or testing in the longitudinal direction utilising uniaxial tensile testing. Due to the limited volume of transverse sinus tissue obtainable from the porcine dura mater, the transverse sinus tissue was only evaluated in the circumferential direction using ring testing.

Circumferential test samples were cut into strips of width $\approx$ 2–3 mm in preparation for ring testing. Longitudinal vessel specimens of $\approx$ 30 mm length were dissected from the 3 segments of the SSS (the frontal, parietal and occipital regions). Note that to ensure appropriate sample dimensions for pure tension testing (a specimen length to width ratio greater than 5:1^39^), longitudinal samples were tested in their native tubular configuration (i.e. they were not ’splayed open’ with a longitudinal incision prior to characterisation). See Fig. S1 of Supplementary information for illustration of test directions and sample geometries. Due to limited tissue volume, each region of the SSS was designated to either longitudinal or circumferential direction testing. The mechanical testing samples were stored in phosphate buffer solution (PBS) at $4 \,^{\circ }$C prior to testing. All mechanical testing was carried out within 24 h of tissue thawing.

### Measurement of geometrical properties

Sample cross-sectional area was determined pre-test using noncontact photogrammetry methods. Optical methods have been employed previously to determine the cross-sectional area of parasagittal bridging veins^21^ and of bulk dura mater tissue^40^. For longitudinal test samples, 2 images of the test specimens were acquired at the region of deformation (i.e. at the centre of the specimen’s length) from different viewpoints. One image was captured from above the centre of the test sample (i.e. from a top view), while the second image was acquired from a side view of the sample centre. The top view of the test sample allowed for calculation of the sample width while the side view enabled assessment of the sample thickness. 3 measurements were then manually acquired from each of the 2 images utilising ImageJ’s ’measure’ function to provide an average thickness and width at the sample centre. Circumferential test samples were imaged in the ring test setup to evaluate the specimen thickness. The samples were imaged under a 0.05 N preload^41,42^ to ensure samples were not slack during imaging and to provide consistency between sample measurements. Similar to longitudinal test specimens, images of the specimen centres were acquired from both a top and side view. Sample dimensions were then manually recorded utilising ImageJ to evaluate any region-specific anatomical variations.

### Measurement of mechanical properties

A dedicated biological tissue testing device, the CellScale Biotester (CellScale, Canada) was used to evaluate the sample mechanical properties. Prior to mechanical characterisation, longitudinal test samples were speckled with a specialised biological tissue dye (CDI tissue staining dye) using an airbrush (ABEST airbrush) to facilitate post-test local sample strain analysis utilising digital image correlation (DIC)^43^ (see Fig. S2 of Supplementary information for illustration of DIC protocol). DIC analysis of longitudinal specimens was conducted to account for inaccuracies between local sample strains and clamp deformation, which are primarily due to the effects of sample slippage from the clamps^44^. Cellscale’s Labjoy software was utilised to conduct the DIC analysis of the longitudinal test specimen experimental images. A user-defined grid was first applied over the initial, undeformed image of the test specimens. The grid was applied to the central region of the test samples to ensure the strain concentrations associated with sample gripping did not interfere with strain measurements^45^. The software then calculates the deformation within subsections of the initial grid and determines the local nominal strain in the central region of the specimen. To calculate the sample stretch ratio, the nominal strain values across the DIC grid were averaged at each time point to provide an average nominal strain ($\epsilon$) in the central region of the test samples. The stretch ratio values ($\lambda$) for the experiments were then calculated using the following equation:

1 $$\begin{aligned} \lambda = 1 + \epsilon \end{aligned}$$

Stretch ratio was employed as it is used extensively in soft biological tissue deformation measurement^42^.

Ring test specimen deformation was instead analysed using global stretch measurements i.e. based on the displacement of the ring testing pins. At large deformations, pin displacement becomes linear with local ring sample deformation and global measurements thus provide an average measure of the stretch ratio along the ring sample circumference^46^ and importantly, do not suffer from the effects of sample slippage encountered in tensile testing. Speckling of the ring specimen’s cross-section for DIC was attempted but was not possible due to the structure’s limited wall thickness^46^.

Longitudinal tensile testing was conducted in pure tension as the length to width ratio of test samples was >5:1 when samples were secured in test clamps^42^. Ring testing was conducted utilising specialised metal pins ($\approx$ 1 mm diameter) (see Fig. S2 of Supplementary information for illustration of mechanical testing setup for both circumferential and longitudinal testing). Uniaxial tensile test samples were subjected to a preload of 0.1 N while ring samples were preloaded to only 0.05 N to account for their smaller cross-sectional area. Preload was applied to account for the residual stresses inherent to excised vascular tissues and to mimic the in vivo residual strain environment^42^. 10 cycles of sample preconditioning were then conducted to a stretch ratio of $\lambda$ = 1.03 at a displacement rate of 1% of the sample gauge length per second. Samples were then stretched to failure for tissue characterisation. Venous^47^ and dura mater tissues^40^ can be considered incompressible materials due to their inherent high water content. Thus, the Cauchy stress for deriving the Cauchy stress-stretch curves was calculated based on the pre-test cross-sectional area measurement^48^. To compare the stiffness of samples between the different regions of interest and for the different test directions, an elastic modulus (E_stiff_) was calculated through the application of a linear fit to the high stiffness, linear domain of the Cauchy stress-stretch curves of each sample^25,34^. All linear fits of had coefficient of determination (R^2^) values > 0.98^34^.

### Measurement of structural properties

#### Histological staining

Histological staining samples were fixed in 2% glutaraldehyde and 2% paraformaldehyde in PBS buffer at $4\,^{\circ }$C overnight to induce chemical fixation. Samples were then embedded in OCT compound and snap-frozen in liquid nitrogen. The frozen samples were cryosectioned into 15 $\upmu$m-thickness slices using a Leica cryostat (Leica CM1860 UV) and stained using an elastic stain kit (Verhoeff–Van Gieson, Abcam ab150667) for visualisation of collagen, elastin and smooth muscle. Specimens were observed under an optical microscope at 4 *x* magnification and images were then stitched using Adobe Photoshop Lightroom Classic (Version 8.4.1) to produce an image of the whole sinus cross-section. The images were then processed using a colour deconvolution plugin^49^ as has been utilised previously on a host of biological tissues^50–52^. The area fraction of collagen and smooth muscle tissue was determined from the deconvolved images by utilising ImageJ to determine the ratio of stained constituent pixels to the total number of pixels representing each sample^53^. For further details on the colour deconvolution and area fraction analysis protocols please refer to the Supplementary methods in the Supplementary information file.

#### Scanning electron microscopy (SEM)

SEM specimens were first chemically fixed in a solution of 2% glutaraldehyde in PBS buffer for 4 h followed by immersion in 2% glutaraldehyde and 2% paraformaldehyde in PBS buffer overnight. Both steps were carried out at 4 $^{\circ }$C and both buffers contained 1% antimicrobial (containing both antibiotics and antifungals) to prevent microbial contamination of samples. Samples were then rinsed in PBS solution followed by immersion in a tissue maceration buffer (1 N NaOH solution with 1% antimicrobial solution) for 1 week at room temperature. The 1 N NaOH maceration method has been employed previously to study the collagenic architecture of dura mater tissue^54^ and is used to remove all noncollagenic components of soft biological tissues^55^. The maceration buffer was changed daily to remove the digested tissue. Following the 1 week of maceration, the samples were placed in PBS solution with antimocrobials for 3 days to remove any remaining digested tissue. The samples were then dehydrated in graded alcohol and dried; first for 20 minutes in a 1:1 solution of Hexamethyldisilizane (HMDS):100% ethanol and then for 20 minutes in a 100% HMDS solution^56^. A small layer of 100% HMDS was then left covering test samples and left to evaporate overnight in a fume hood to prevent the sample deformation associated with air drying^56^.

### Statistical analysis

Statistical analyses were conducted with Prism software (GraphPad Prism 8.2.1). Data are represented as a mean ± standard error about the mean. The normality of all test variables was analysed using Shapiro-Wilk tests. An ordinary one-way analysis of variance (ANOVA) test was used to compare the averaged longitudinal test sample E_stiff_ values of the different test groups. The averaged circumferential test sample E_stiff_ values were found to have not normal distributions and so the Kruskal–Wallis test was used to compare the different groups. The Mann–Whitney test was utilised to compare the stiffness of the SSS in the longitudinal and circumferential test directions, independent of the test region within the SSS. A *p* value (alpha value) < 0.05 was considered statistically significant for all statistical tests.

## Results

### Mechanical properties

**Figure 2:**
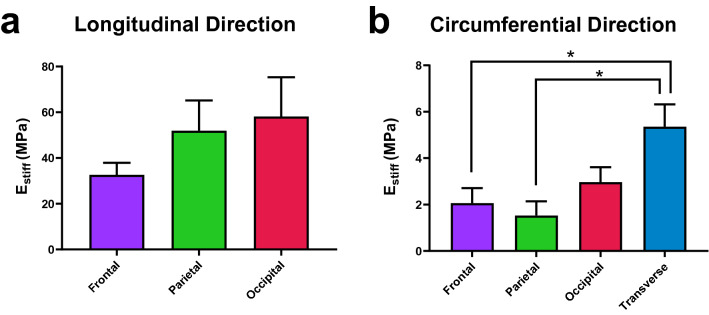
(**a**) Mean E_stiff_ results for the samples tested in the longitudinal test direction utilising uniaxial tensile testing. (**b**) Mean E_stiff_ results for the samples tested in the circumferential test direction utilising ring testing. *Statistical significance ($p< 0.05$), error bars represent standard error about the mean. Note that the y-axis scale differs between (**a**) and (**b**).

The Cauchy stress-strain curves for the samples tested in the longitudinal direction utilising uniaxial tensile testing and in the circumferential direction utilising ring testing are shown in Fig. 1a,b, respectively. Note that the transverse sinus was only tested in the circumferential test direction due to the limited volume of tissue which could be excised from this region. The J-shaped curve of the dural venous sinus tissue is evident in the Cauchy stress-strain curves of both testing modalities, indicating a hyperelastic tissue. E_stiff_ values were calculated for the various regions to allow for evaluation of any regional dependence of this parameter^25^. The E_stiff_ values were calculated utilising a Matlab algorithm in which a linear fit was applied to the high stretch region of the Cauchy stress-stretch curves^34^. All linear fits had an R^2^ value > 0.98 and were thus representative of the experimental stress-stretch data^34,57^. The E_stiff_ values are listed in Table 1 and statistical comparisons are conducted in Fig. 2.

**Table 1:** E_stiff_ values for both the longitudinal and circumferential test directions, along with the number of tests conducted per region and testing direction.

Region	Longitudinal (MPa)	Longitudinal tests conducted	Circumferential (MPa)	Circumferential tests conducted	Lumen diameter (mm)
Frontal	32.6 ± 5.3	5	2.1 ± 0.6	9	2.9 ± 0.4
Parietal	51.9 ± 13.3	5	1.5 ± 0.6	9	4.1 ± 0.2
Occipital	58.1 ± 17.2	5	3.0 ± 0.7	9	5.1 ± 0.6
Transverse	–	–	5.4 ± 1.0	10	3.9 ± 0.4

Note that while 10 tests were conducted for the transverse region in the circumferential direction, 1 test was discarded due to hardware issues. Also shown are the lumen diameter values for the various regions. Data are represented as a mean ± standard error about the mean.

#### Porcine dural venous sinus tissue exhibits regional and directional anisotropy

Regional differences in E_stiff_ results in the samples tested in the circumferential direction were identified, with the transverse sinus having a statistically significantly higher stiffness than both the frontal and parietal regions of the SSS (Fig. 2b). A trend of increasing stiffness from anterior to posterior was observed in the longitudinal test direction (Fig. 2a), however, these differences did not prove statistically significant, possibly due to the high inter- and intra-biological variance observable in dura mater tissue^34,40^. Statistically significant anisotropic behaviour was observed in the SSS mechanical testing results through comparison of the longitudinal and circumferential test results, independent of test region within the SSS, utilising a Mann–Whitney statistical test ($p<0.0001$).

### Structural properties

**Figure 3:**
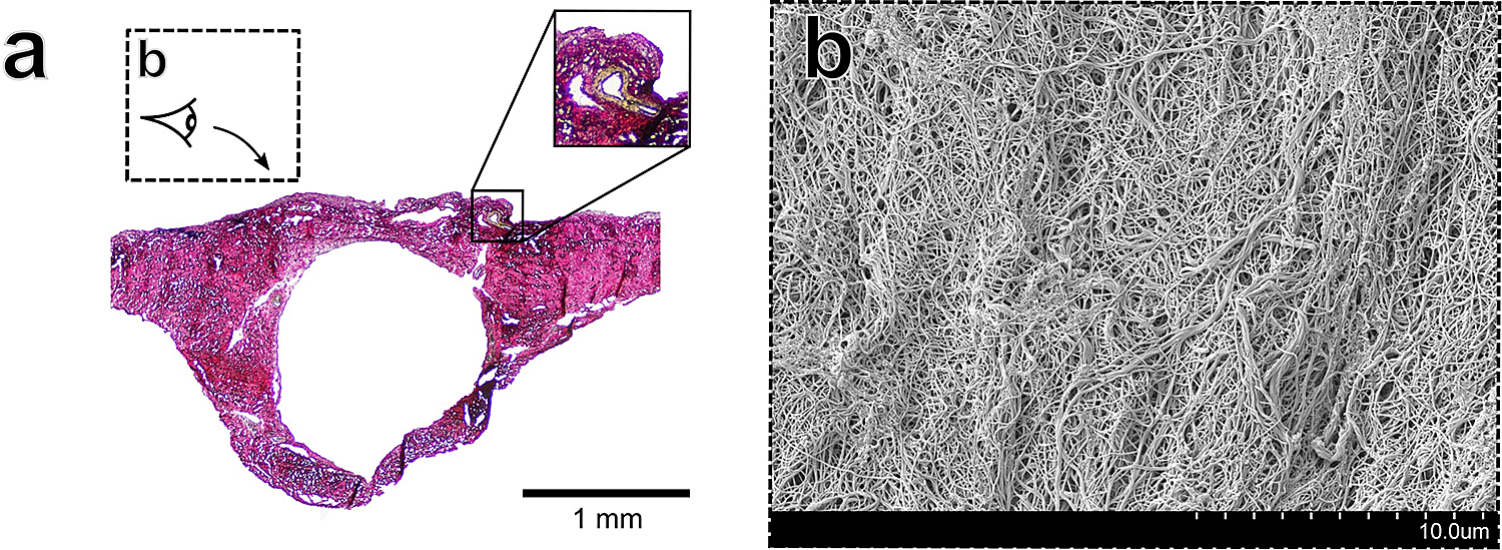
Structural images of the frontal region of the SSS (**a**) Verhoeff–Van Gieson stained sample of the frontal region. The solid-line inset image shows what appears to be a meningeal artery (**b**) SEM imaging of the bone surface layer (as illustrated in dashed-line inset of (**a**)) showing disorganised focal aggregations of collagen fibres.

**Figure 4:**
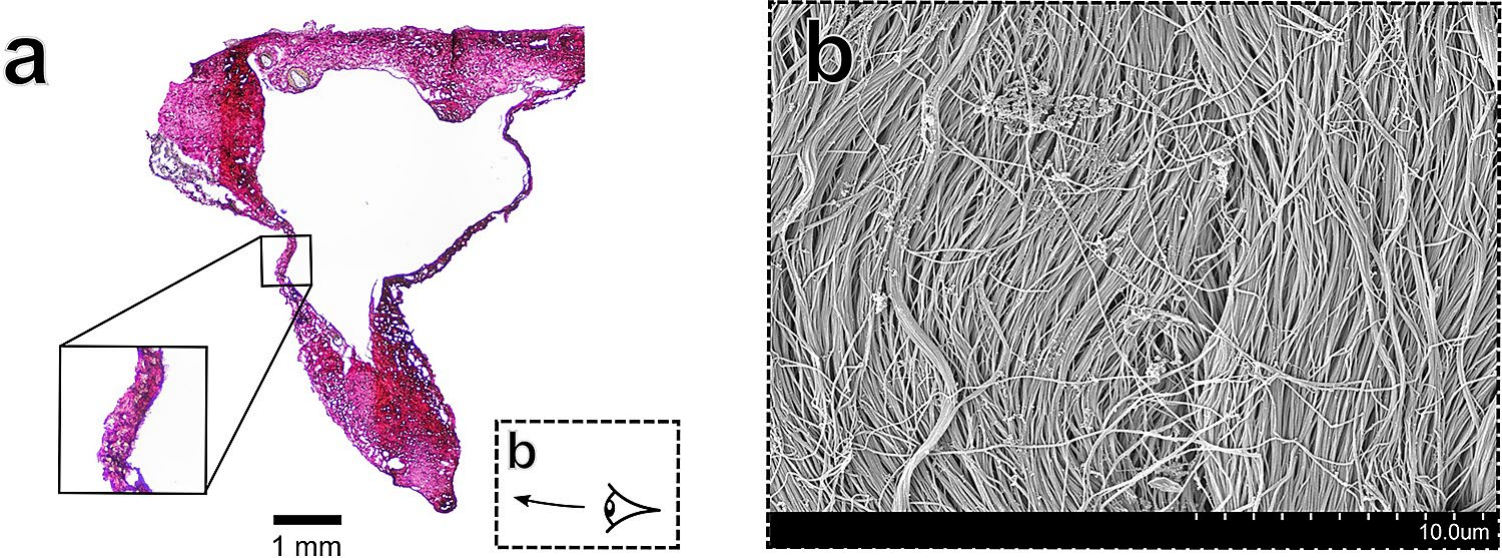
Structural images of the parietal region of the SSS (**a**) Verhoeff–Van Gieson stained sample. The solid-line inset image shows the thin, collagenic wall of the sinus with a thin endothelial layer on the lumen wall (**b**) The arachnoid surface of the sinus (as illustrated in dashed-line inset of (**a**)) demonstrating collagenic alignment along the longitudinal direction of the sinus.

**Figure 5:**
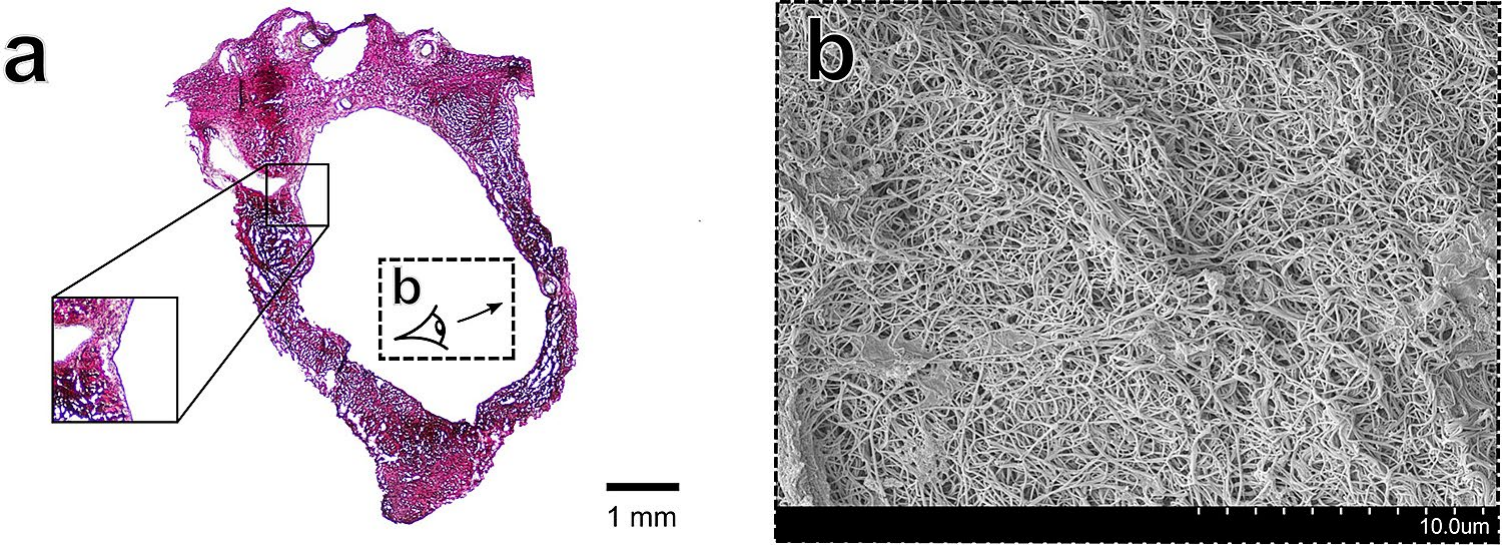
Structural images of the occipital region of the SSS (**a**) Verhoeff–Van Gieson stained sample. The solid-line inset image again demonstrates the thin endothelial later of the dural sinus tissue (**b**) The surface of the occipital region lumen wall (as illustrated in dashed-line inset of (**a**)) depicting seemingly random collagenic alignment, with the endothelial layer removed from the maceration protocol.

**Figure 6:**
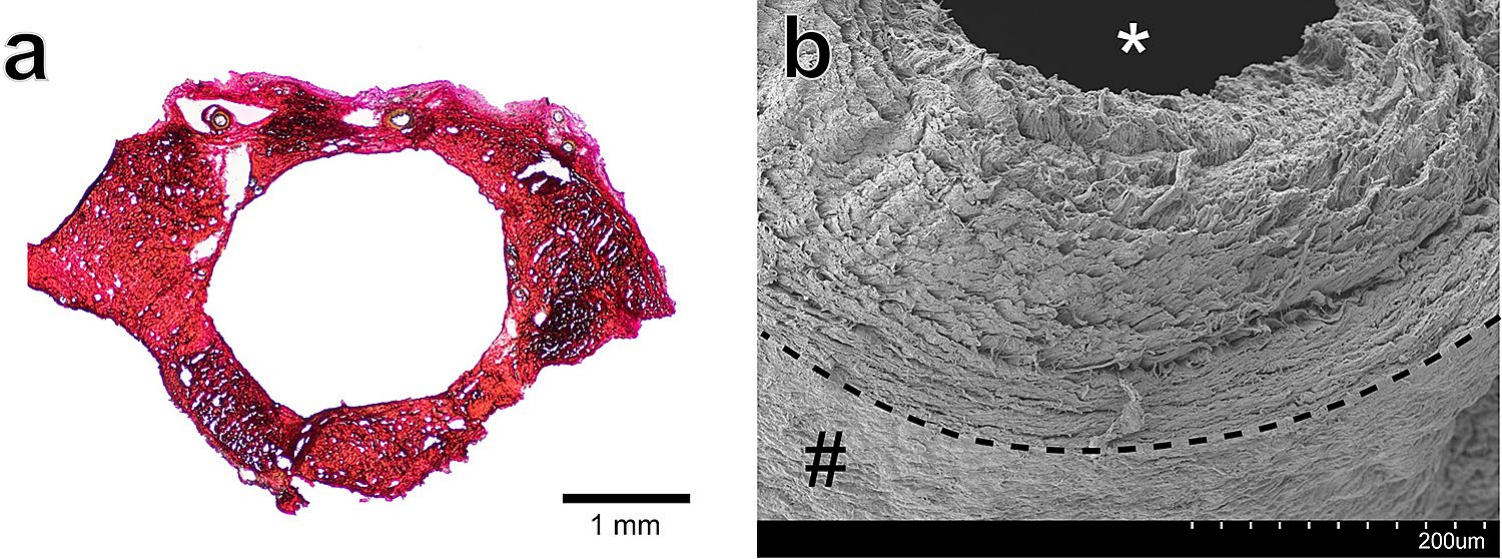
Structural images of the transverse sinus (**a**) Verhoeff–Van Gieson stained sample. (**b**) The cross-section of the dural sinus wall with the lumen (*) and arachnoid surface (#) highlighted. Distinct layers appear to constitute the wall of the dural sinus tissue.

Utilisation of Verhoeff–Van Gieson histological staining allowed for visualisation of the collagen, elastin and smooth muscle of the sinuses (Figs. 3, 4, 5, 6 part (a)). The area fraction (AF) percentages of collagen and smooth muscle for each region are listed in Table 2. The inset of Fig. 3a depicts what appears to be a meningeal artery, with the smooth muscle tissue of the arterial wall stained yellow with the Verhoeff–Van Gieson stain. The dense microvascular network of arteries within the walls of the dural sinuses has been studied previously^58^. Larger meningeal arteries (with a diameter of 400 to 800 micron) divide into a rich, tortuous network of anastomotic vessels^59^. These vessels supply nutrients to the inner table of the cranial vault and to the dura mater tissue^59^. A thin endothelial layer could be observed in all histological images of the sinuses (see solid line insets of Figs. 4 and 5).

#### Porcine dural venous sinus tissue has a large collagen content and exhibits regional variations in smooth muscle content

Utilising area fraction analysis, the biochemical basis of the dural sinuses mechanical anisotropy was investigated. Quantitative analysis of the area fractions of fibrous collagen and smooth muscle tissue constituents was carried out utilising a colour deconvolution protocol^49^ to gain insight into the region-dependent mechanical properties observed. Constituent analysis demonstrated the large fibrous collagen content of the sinuses (with area fractions ranging from $\approx$ 84 to 94%), while smooth muscle area fraction analysis identified region-dependent variations in smooth muscle content (Table 2). In particular, the transverse sinus and the occipital region of the SSS exhibited higher smooth muscle area fractions (49.6 ± 7.2 $\%$ and 33.4 ± 9.4 $\%$, respectively) when compared to the frontal and parietal regions (22.7 ± 4.4 $\%$ and 24.8 ± 5.4 $\%$, respectively). However, no statistical significance was identified between regions in either the collagen or smooth muscle area fractions.

#### Porcine dural venous sinus tissue exhibits layer-specific collagenic alignment

The 1 N NaOH maceration method allowed for visualisation of the collagenic architecture of the sinuses as shown in Figs. 3, 4, 5 and 6 part (b). Random alignment was observed on both the the bone surface (Fig. 3b) and the vessel lumen surface (Fig. 5b) of the sinuses. In contrast, layer-specific collagenic alignment was observed on the arachnoid surface of the sinuses (i.e. the external surface of the sinus that is adjacent to the arachnoid mater membrane as described previously^54^) along the longitudinal direction of the vessels (Fig. 4b). This is also in contrast to the alignment observed on the arachnoid surface of the human dura, which has been demonstrated to have random collagenic alignment on both the bone and arachnoid surfaces of the tissue^54^. Similar to the findings of a dura mater collagenic architecture investigation, distinct layers appear to be observable in the cross-section of the transverse sinus as shown in (Fig. 6b)^54^.

### Geometrical properties

#### The porcine SSS diameter increases anterior to posterior

Accurate geometric modelling of the various tissues of the head is a key parameter for accurate TBI model outputs^19,20^. As shown in Table 1, the mean diameter of the SSS lumen increased anterior to posterior from 2.9 mm in the frontal region to 5.1 mm in the occipital region. The transverse sinus in comparison exhibited a mean lumen diameter of 3.9 mm. The identified anatomical variations in SSS diameter observed in this investigation have been observed previously in human subjects in magnetic resonance imaging study^60^.

## Discussion

To address the paucity of material characterisation conducted on the dural venous sinuses, in this study we investigate, for the first time, the mechanical, structural and geometrical properties of the porcine dural venous sinus tissues, structures which we hypothesise may play a role in influencing brain strains during TBI and TBI-mediated ASDH.

FE modelling of head injuries has provided the TBI research community with important insights into the fundamental mechanopathological mechanisms of head impacts. In order to aid the predictive capacity of head impact simulations, biofidelic modelling of the native head tissues’ mechanical and structural properties is of key importance^19^. The anatomical connection between the largest of the dural sinuses, the SSS, and the falx cerebri, is of particular interest when attempting to computationally predict TBI brain strains. Most FE head models which include the dural sinuses assume that these structures have mechanical properties identical to that of native dura mater tissue^12,61,62^, despite a lack of evidence supporting this assumption. Specifically, an elastic modulus of 31.5 MPa is assigned to the sinus tissues, based on the mechanics of native human dura mater tissue^63^. Our previous work identified, for the first time, potential mechanical and structural differences between dura mater tissue and the SSS^34^. Here, we demonstrate that the dural venous sinuses have mechanical properties which appear to differ from that of native dura mater tissue. Native porcine dura mater tissue exhibited E_stiff_ values ranging from $\approx$ 8 to 16 MPa^34^, while the porcine dural sinuses tested in this investigation had E_stiff_ values ranging from $\approx$ 33 to 58 MPa in the longitudinal test direction (Table 1). However, it is worth noting that our previous investigation on native dura mater tissue did not incorporate DIC analysis, which could potentially lead to an underestimation of the tissue’s mechanical stiffness^44^. Still, even with these potential experimental variations considered, the results presented in this investigation suggest that the dural venous sinuses have a higher stiffness than native dura mater tissue. Thus, the dural sinuses appear to be mechanically inhomogeneous with native dura mater tissue, which may have implications in the study of falx-induced brain strains.

ASDH, a common co-morbidity of severe TBI, is frequently caused by rupture of parasagittal bridging veins which drain into the SSS^23^. Mechanical mismatches between the connected tissues may result in strain concentrations at the intersection between the tissues, thus potentially increasing the propensity of bridging veins to haemorrhage. Previous analysis of bridging vein mechanics in mature porcine subjects identified that the bridging veins exhibited a mean E_stiff_ value of $\approx$ 22 MPa in the vessels longitudinal direction (at moderately low strain rates)^21^. A study on the bridging vein distribution in human cadavers has shown that most of the parasagittal veins drain into the frontal and parietal regions of the SSS^64^. Comparing the mechanics of the porcine bridging veins to the frontal and parietal regions of the porcine SSS, these regions exhibited E_stiff_ values of $\approx$ 33 and 52 MPa, respectively, in the longitudinal test direction (see Table 1), significantly larger than the value of $\approx$ 22 MPa identified for porcine bridging veins^21^. The potential E_stiff_ mismatch between bridging veins and the dural sinuses they drain into suggests that strain concentrations may occur at the junction between these tissues during dynamic loading. These findings warrant further investigation for improving the predictive capacity of FE models of ASDH.

The results of this investigation also provide the first evaluation of the sinus’ circumferential mechanical behaviour. The circumferential direction mechanics of the porcine dural sinus tissue, which can provide insights into inflation behaviour, indicated that the tissue has elastic moduli ranging from $\approx$ 1.5 to 5.5 MPa (see Table 1). Previously, a study on the mechanics of porcine jugular veins, which also employed ring testing, demonstrated that the high strain region moduli of porcine jugular veins in the circumferential direction were $\approx$ 19 MPa^65^. Similarly, an investigation on porcine vena cava vessels, which again employed ring testing, showed that the high strain region moduli in the circumferential direction of the vena cava were $\approx$ 14 MPa^66^, again significantly larger than than that of the dural venous sinuses. The significantly smaller moduli observed in the dural sinuses, when compared to that of other venous structures in the same species, suggests that significant consideration should be given to the inflation pressure applied during dural sinus angioplasty procedures in order to prevent permanent tissue damage^67^. Furthermore, given that microcatheter balloons designed for coronary angioplasty procedures are employed in the treatment of sinus pathologies^32^, the results presented herein can be utilised to inform the design of prospective microcatheter devices specifically for the dural sinuses.

The hemodynamic and biomechanical forces tissues are exposed to in vivo play a key role in modulating the alignment and distribution of key structural proteins such as collagens^68^. Investigations on other vascular tissues’ collagenic alignment have demonstrated that collagen fibres are aligned primarily in the longitudinal direction of blood vessels and thus, primarily contribute to longitudinal vessel stiffness^69^. This longitudinal alignment, also observed herein on the dural sinus arachnoid surface (see Fig. 4b), may explain the large longitudinal stiffness observed in the dural sinus tissue. In relation to the regional mechanical properties, the SSS trended towards a higher elastic modulus moving anterior to posterior in both the circumferential and longitudinal test directions (see Fig. 2). Smooth muscle cells are posited as playing an important role in mediating vessel circumferential tension in vivo^70^ and regional variations in smooth muscle content were observed in this study (see Table 2). However, while smooth muscle cells can contract and contribute to in vivo vessel mechanics, they become inactive in excised vascalature^71^. Thus, the smooth muscle content of the dural sinus tissue likely has a negligible effect on the tissue’s passive, ex vivo mechanical response. The reason for the regional anisotropy may instead pertain to regional variations in haemodynamic conditions in vivo. The SSS is responsible for draining the majority of venous outflow from the cerebral hemispheres^72^. Blood flows posteriorly within the SSS, with flow from venous tributaries along its length leading to increased volumes of blood as the flow runs anterior to posterior. The increased shear stress associated with the increased volume of flow in the posterior sections of the SSS, and the resulting variation in biomechanical remodelling, could thus explain the regional mechanical variations observed in the SSS mechanics^68^.

**Table 2:** Collagen area fraction (AF) values and smooth muscle area fraction (AF) values for the various regions of the dural venous sinuses.

Region	Collagen area fraction (AF) (%)	Smooth muscle area fraction (AF) (%)
Frontal	84.8 ± 3.4	22.7 ± 4.4
Parietal	84.5 ± 3.9	24.8 ± 5.4
Occipital	84.5 ± 4.2	33.4 ± 9.4
Transverse	94.0 ± 1.1	49.6 ± 7.2

Data are represented as a mean ± standard error about the mean.

Compositional analysis of the porcine dural sinuses revealed collagen area fractions ranging from $\approx$ 84 to 94%. Previous collagen area fraction analysis of human SSS identified a mean collagen fibre area fraction of $\approx$ 60% for the SSS tissue^73^, significantly lower than the results presented herein. There are a number of potential explanations for this discrepancy. First, the authors of the previous investigation do not state the thickness of the sectioned slices on which histological analysis was conducted. The thickness of the sectioned slices influences the extent of co-localisation within stained tissue sections and thus will affect the results obtained from area fraction analysis. Furthermore, the aforementioned investigation utilises an automatic pixel labelling approach to identify the area fractions within stained images^73^. Studies have shown variations in results obtained from differing stain segmentation approaches^74^ and this may also contribute to the discrepancies in the results between the studies. Nonetheless, the dural sinuses are a highly collagenous, stiff tissue and their biomechanical behaviour warrants further consideration in the study of neuropathologies such as TBI and CVST.

There are a number of limitations related to this study. Firstly, the porcine tissue evaluated herein may not accurately represent the behaviour of human dural sinus tissue. However, a comprehensive interspecies comparison of the cerebral venous system has indicated that the the SSS and transverse sinus are anatomically comparable across numerous species including rats, sheep and humans^75^. Moreover, numerous similarities have been identified between the pig and human neurovascular systems and thus, pigs have long been used as animal models for various cerebral venous pathologies^76^. However, the porcine dura mater does not contain a fully-formed falx^77^. Therefore, for improved translatability of results, future work will focus on replicating the analysis presented herein on human dural venous sinus tissue. The results presented in this study are based on immature porcine specimens ($\approx$ 7 months old). Age-dependent mechanical variations have been observed previously in human dura mater tissue^78^ and porcine brain tissue^79^. While evaluating age-related effects was outside the scope of this study, future studies should investigate the potential age-dependency of dural sinus mechanical and structural properties. High biological variance was observed for the SSS specimens tested in the longitudinal direction. High inter- and intra-biological variance has been observed previously in native dura mater tissue^34,40^. While a relatively small number of samples were tested in the longitudinal direction (5 for each region of the SSS), the variance observed is comparable to previous investigations of human dura mater tissue^11,80,81^. However, future studies should focus on a larger number of experimental replicates to account for the large biological variance observed in dura mater tissue. Finally, while the quasi-static strain rate utilised in this investigation is likely representative from the perspective of studying vessel response to angioplasty inflation^82^, the strain rate may not accurately capitulate the response of the dural sinuses in highly dynamic events such as TBI and TBI-mediated ASDH. TBI events result in strain rates ranging from $\approx$ 30 up to 90/s^83^, which is orders of magnitude higher than the quasi-static strain rate employed in this investigation. However, the mechanical properties described in this study serve as a useful guide given the dearth of properties in the literature. Future investigations should focus on investigating the rate-dependent behaviour of the dural sinuses along with the falx and tentorium.

## Conclusions

This work represents the first mechanical and structural characterisation of the dural venous sinuses. Mechanical characterisation of the sinuses identified regional and directional stiffness differences in the tissues. The transverse sinus demonstrated higher mechanical stiffness that the regions of the superior sagittal sinus and samples tested in the longitudinal direction exhibited higher mechanical stiffness than those tested in the circumferential direction. Superior sagittal sinus specimens tested in the longitudinal test direction also exhibited higher stiffness than that of native porcine dura mater tissue, which has important implications for the biofidelity of current FE head models. Biochemically, histological staining identified that the transverse region had a higher area fraction of collagen than the regions of the superior sagittal sinus, which may explain the larger mechanical stiffness of this region. The findings in this investigation can aid in the efficacy of mechanical thrombectomy procedures for improving sinus patency and have the potential to further improve the biofidelity and injury prediction accuracy of TBI computational models. Future work will focus on replicating this analysis on human cadaveric tissue for improved translatability of results.

## Supplementary Information

Supplementary Information.
