# Supplementary material for: Mechanical and structural characterisation of the dural venous sinuses
Source: Sci Rep. 2020 Dec 10;10:21763. doi: 10.1038/s41598-020-78694-4 (PMC7729903; doi:10.1038/s41598-020-78694-4)
Supplement: Supplementary file 1 — Supplementary Information. [file 41598_2020_78694_MOESM1_ESM.pdf]

Supplementary Information: Mechanical and Structural Characterisation of the Dural Venous Sinuses

Darragh R. Walsh, James J. Lynch, David T. O' Connor, David T. Newport, John J.E. Mulvihill

Materials and Methods

Measurement of mechanical properties

The testing directions and sample geometries for mechanical characterisation are as described in Fig. S1.

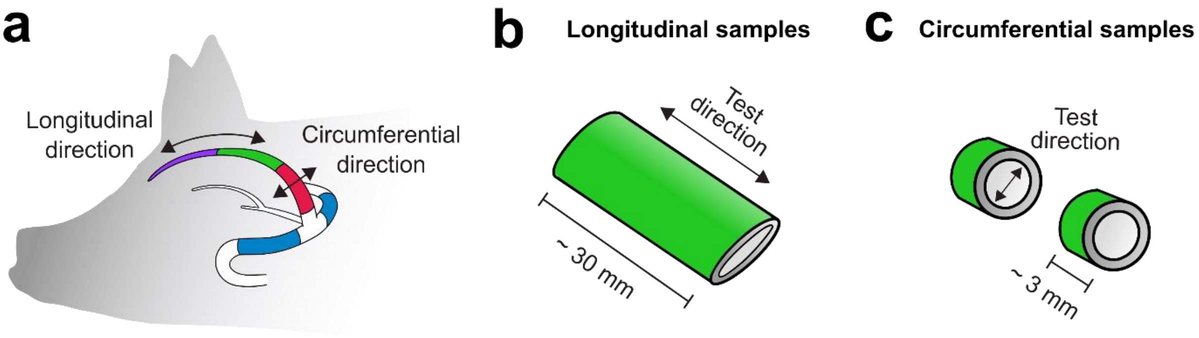

Figure S1. (a) Illustration of the testing directions selected for mechanical characterisation. (b) Longitudinal sample geometry and mechanical test direction. (c) Circumferential sample geometry and mechanical test direction.

The mechanical testing workflow for both the longitudinal and circumferential testing directions are as illustrated in Fig. S2.

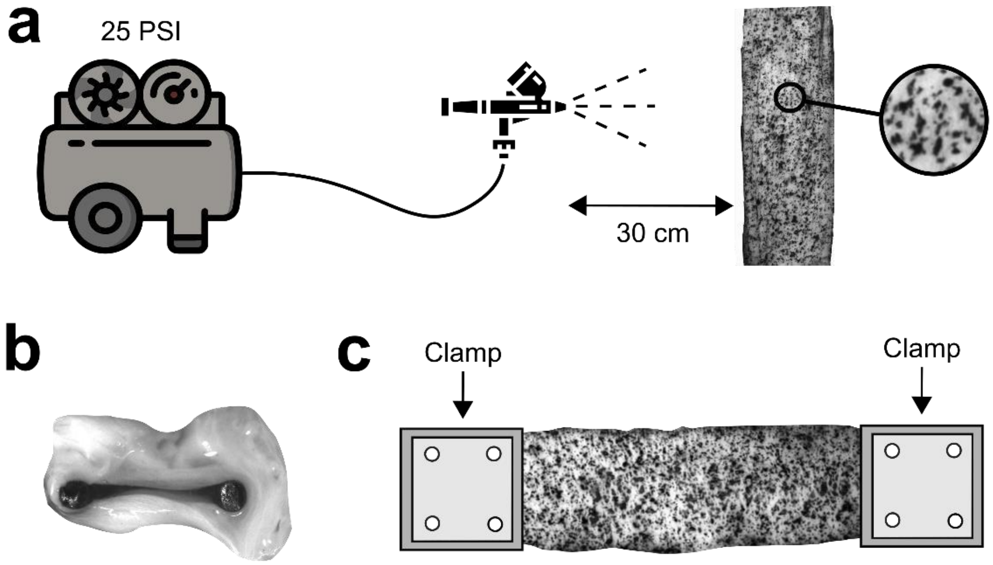

Figure S2. (a) Digital image correlation protocol. The airbrush compressor pressure was set to 25 PSI and the nozzle was held 30 cm from the test sample. (b) Circumferential test sample in the ring test configuration. (c) Illustration of the longitudinal test configuration.

Colour deconvolution and area fraction analysis protocol

For optimisation of the colour deconvolution algorithm, a subset of microtomed tissue sections were stained utilising single-stains only. This allowed for accurate quantification of the individual dye red/green/blue (RGB) values prior to colour devonvolution, without the erroneous effects of colocalisation associated with more than one dye staining a tissue section. The RGB values for the individual tissue constituents are as described in Table S1.

Tissue Component	Decimal Code (R,G,B)
Collagen	(5,247,64)
Elastin	(150,158,133)
Smooth Muscle	(15,84,240)

Table S1. The decimal code RGB values for the individual tissue components identified from the single stain histological images.

These decimal code values can then be utilised to deconvolve the tissue components in Van-Gieson histologically stained samples using a colour deconvolution algorithm (see Fig. S3 for colour deconvolution example).

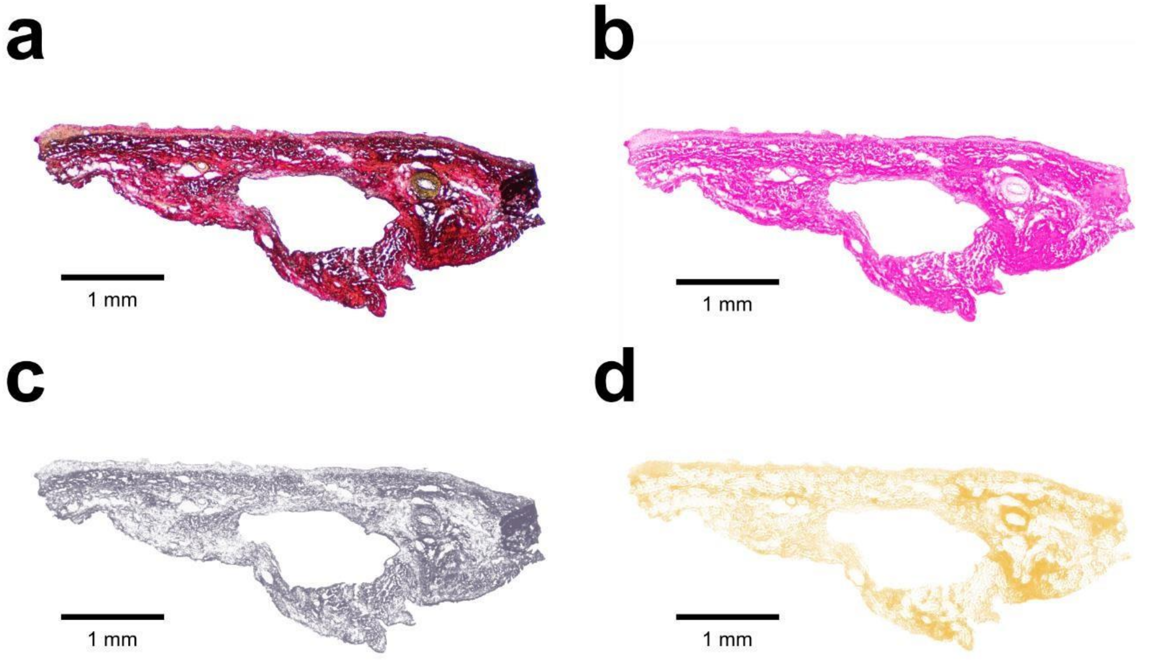

Figure S3. Application of the colour deconvolution algorithm to a Van-Gieson stained dural sinus sample. (a) Histological image of a Van-Gieson-stained sample from the frontal region of the porcine dural sinus. (b) Deconvolved collagenic component of the original image. (c) Deconvolved elastin component of the original image. (d) Deconvolved smooth muscle component of the original image.

For area fraction analysis, the total number of pixels stained by the Van-Gieson stain was first determined. The Van-Gieson stained images (example shown in Fig. S3 (a)) were processed with the 'colour threshold' plugin in ImageJ. The number of pixels in the colour thresholded images were then tabulated using the 'Measure' tool in ImageJ. To determine the number of pixels occupied by the various tissue components, the deconvolved images (Fig. S3 (b-d)) were also thresholded using the ImageJ Threshold tool. Again, the number of pixels in the thresholded images were calculated using the 'Measure' tool in ImageJ. The area fraction of each tissue component was then calculated by determining the ratio of constituent pixels to the total number of pixels. However, the elastin tissue component was excluded from analysis due to potential false positive identification of this component with the cellular nuclei staining of the Van-Gieson stain. The Van-Gieson stain stains nuclei blue to black, which has an RGB profile similar to that of the elastin dye. Therefore, only the collagen and smooth muscle area fractions were analysed and quantified.
